# Reduced Odds of Mpox-Associated Hospitalization Among Persons Who Received JYNNEOS Vaccine — California, May 2022–May 2023

**DOI:** 10.15585/mmwr.mm7236a4

**Published:** 2023-09-08

**Authors:** Samuel Schildhauer, Kayla Saadeh, Josh Vance, Joshua Quint, Tarek Salih, Timothy Lo, Awa Keinde, Edwin Chojolan, Esther Gotlieb, Marisa Ramos, Eric Chapman, Philip Peters, Jessica Watson, Kelly A. Johnson, Eric C. Tang, Kathleen Jacobson, Robert Snyder

**Affiliations:** ^1^California Department of Public Health, Richmond and Sacramento, California; ^2^Immunization Services Division, National Center for Immunization and Respiratory Diseases, CDC; ^3^Division of HIV Prevention, National Center for HIV, Viral Hepatitis, STD, and TB Prevention, CDC.

**Summary**

**What is already known about this topic?**

One JYNNEOS vaccine dose decreases mpox lesion severity and hospitalization prevalence.

**What is added by this report?**

Among persons with and without HIV infection, mpox-associated hospitalization rates were lower among those who had received ≥1 dose of JYNNEOS vaccine compared with those who were unvaccinated.

**What are the implications for public health practice?**

Receiving ≥1 dose of JYNNEOS vaccine reduced the odds of hospitalization among California residents. To maximize enduring immunity against *Monkeypox* *virus* infection, all eligible persons should complete the 2-dose JYNNEOS vaccine series.

## Abstract

The effectiveness of 1 dose of JYNNEOS vaccine (modified vaccinia Ankara vaccine, Bavarian Nordic) against hospitalization for mpox (caused by *Monkeypox virus*), has been demonstrated; however, the impact of 2 doses on hospitalization risk, especially among persons infected with HIV, who are at higher risk for severe disease, is an important factor in evaluating vaccine effectiveness against mpox disease severity and *Monkeypox* *virus* infection. Surveillance data collected by the California Department of Public Health were used to evaluate whether receipt of 2 doses of JYNNEOS vaccine reduced the odds of hospitalization among persons with mpox. The odds of hospitalization among persons with mpox who had received 1 or 2 JYNNEOS doses were 0.27 (95% CI = 0.08–0.65) and 0.20 (95% CI = 0.01–0.90), respectively, compared with unvaccinated mpox patients. In mpox patients with HIV infection, the odds of hospitalization among those who had received 1 JYNNEOS vaccine dose was 0.28 (95% CI = 0.05–0.91) times that of those who were unvaccinated. No mpox-associated hospitalizations were identified among persons infected with HIV who had received 2 JYNNEOS vaccine doses. To optimize durable immunity, all eligible persons at risk for mpox, especially those infected with HIV, should complete the 2-dose JYNNEOS series.

## Introduction

During May 12, 2022–May 18, 2023, a total of 5,765 persons with mpox and 250 (4.3%) mpox-associated hospitalizations were reported among California residents (*1*). At the end of May 2022, California began to distribute JYNNEOS smallpox and mpox vaccine, licensed in the United States as a 2-dose series, with doses administered 28 days apart, for protection against mpox (*2*–*4*). Observational studies of JYNNEOS vaccine effectiveness against mpox have ranged from 66% to 89% for 2 doses and 36% to 75% for 1 dose (*5*–*7*). A 2022 study in 29 jurisdictions found that persons with mpox who had received 1 JYNNEOS dose were less likely to be hospitalized and reported fewer lesions compared with unvaccinated persons with mpox (*8*). The effect of 2 JYNNEOS doses on hospitalization risk, especially among persons with HIV infection, who are at higher risk for severe mpox disease, has not been evaluated (*1*). This study analyzed reported California mpox cases and immunization registry data to determine the risk for mpox-associated hospitalization by JYNNEOS vaccination status.

## Methods

California residents with laboratory-confirmed *Monkeypox* *virus* infections were interviewed to obtain data on demographic, epidemiologic, and clinical characteristics. Case reports with missing information on hospitalization status were excluded. JYNNEOS vaccination status was based on data reported to the California Immunization Registry.* Mpox patients were categorized by vaccination status and HIV status. One-dose recipients were defined as patients who had 1) received 1 preexposure JYNNEOS dose ≥14 days before their episode date,^†^ or 2) received 2 doses, with the second dose administered <14 days before the episode date, or 3) received 2 doses <24 days apart. Two-dose recipients were defined as patients who had received 2 doses ≥24 days apart with the second dose administered ≥14 days before the episode date. Postexposure prophylaxis (PEP) vaccination was defined as receipt of the first JYNNEOS dose after a known or suspected exposure and <14 days before the episode date. Mpox patients who had received zero doses of JYNNEOS vaccine reported to the California Immunization Registry were considered unvaccinated.

Mpox patients with a previous HIV case report in the California Department of Public Health’s Office of AIDS were considered to have HIV infection. Mpox hospitalization was defined as inpatient hospitalization for mpox disease; emergency department visits were not included.

Demographic characteristics of unvaccinated mpox patients were compared with those of 1-dose, 2-dose, and PEP recipients. Odds ratios comparing hospitalization by vaccination status were calculated using binomial logistic regression, and 95% CIs were estimated. Analyses were then stratified by HIV status. To determine whether and how missing hospitalization data might have biased the analysis, mpox patients included in the analysis were compared with those who were excluded because of missing or unknown hospitalization status using Pearson’s chi-square tests. All analyses were conducted using R statistical software (version 4.0.2; R Foundation). This activity was reviewed by CDC and was conducted consistent with applicable federal law and CDC policy.^§^

## Results

Among the 5,765 California residents who had received a diagnosis of mpox, 1,154 (20.0%) were excluded because hospitalization status was not reported. Among the remaining 4,611 included persons, 4,353 (94.4%) were male, 2,083 (45.2%) were Hispanic or Latino, and 3,188 (69.1%) identified as gay, lesbian, or same-gender loving (Table 1). Comparison of patients included in the study and those excluded because of missing hospitalization status identified substantial differences in race and ethnicity, sexual orientation, prevalence of HIV infection, age, and prevalence of missing data on gender identity, race and ethnicity, and sexual orientation (Table 1).

**TABLE 1:** Characteristics of mpox cases, by vaccination status — California, May 12, 2022–May 18, 2023

Characteristic	JYNNEOS vaccination status, no. (%)						p-value^§^
	Received 1 dose n = 230	Received 2 doses n = 79	Received PEP* n = 457	Unvaccinated n = 3,845	Total included N = 4,611	Total excluded^†^ n = 1,154	
**Gender identity**							
Female	0 (—)	1 (1.3)	4 (0.9)	118 (3.1)	**123 (2.7)**	**21 (1.8)**	0.122
Genderqueer or non-binary	4 (1.7)	0 (—)	7 (1.5)	23 (0.6)	**34 (0.7)**	**3 (0.3)**	0.107
Male	223 (97.0)	77 (97.5)	443 (96.9)	3,610 (93.9)	**4,353 (94.4)**	**1,068 (92.5)**	0.021
Transgender female	3 (1.3)	0 (—)	2 (0.4)	51 (1.3)	**56 (1.2)**	**23 (2.0)**	0.058
Transgender male	0 (—)	0 (—)	0 (—)	15 (0.4)	**15 (0.3)**	**4 (0.3)**	1.000
Declined to answer	0 (—)	1 (1.3)	0 (—)	17 (0.4)	**18 (0.4)**	**17 (1.5)**	<0.001
Unknown	0 (—)	0 (—)	1 (0.2)	11 (0.3)	**12 (0.3)**	**18 (1.6)**	<0.001
**Age, yrs, median (IQR)**	35 (22–48)	36 (24.5–47.5)	38 (24–52)	35 (21–49)	**35 (25–48)**	**37 (23–51)**	<0.001
**Race and ethnicity^¶^**							
AI/AN	1 (0.4)	0 (—)	2 (0.4)	18 (0.5)	**21 (0.5)**	**2 (0.2)**	0.272
Asian	17 (7.4)	3 (3.8)	30 (6.6)	200 (5.2)	**250 (5.4)**	**58 (5.0)**	0.644
Black or African American	16 (7.0)	5 (6.3)	32 (7.0)	537 (14.0)	**590 (12.8)**	**108 (9.4)**	0.002
NH/OPI	3 (1.3)	0 (—)	5 (1.1)	14 (0.4)	**22 (0.5)**	**3 (0.3)**	0.451
White	97 (42.2)	31 (39.2)	178 (38.9)	1,023 (26.6)	**1,329 (28.8)**	**333 (28.9)**	1.000
Hispanic or Latino	83 (36.1)	32 (40.5)	175 (38.3)	1,793 (46.6)	**2,083 (45.2)**	**323 (28.0)**	<0.001
Multiple races	3 (1.3)	2 (2.5)	8 (1.8)	61 (1.6)	**74 (1.6)**	**5 (0.4)**	0.003
Unknown	10 (4.3)	6 (7.6)	27 (5.9)	199 (5.2)	**242 (5.2)**	**322 (27.9)**	<0.001
**Sexual orientation**							
Bisexual	9 (3.9)	2 (2.5)	28 (6.1)	424 (11.0)	**463 (10.0)**	**26 (2.3)**	<0.001
Gay, lesbian, or same-gender loving	189 (82.2)	69 (87.3)	375 (82.1)	2,555 (66.4)	**3,188 (69.1)**	**333 (28.9)**	<0.001
Heterosexual or straight	7 (3.0)	2 (2.5)	9 (2.0)	402 (10.5)	**420 (9.1)**	**42 (3.6)**	<0.001
Declined to answer	7 (3.0)	3 (3.8)	10 (2.2)	135 (3.5)	**155 (3.4)**	**21 (1.8)**	0.009
Orientation not listed	6 (2.6)	1 (1.3)	5 (1.1)	63 (1.6)	**75 (1.6)**	**29 (2.5)**	0.057
Unknown	12 (5.2)	2 (2.5)	30 (6.6)	266 (6.9)	**310 (6.7)**	**703 (60.9)**	<0.001
**HIV status**							
Positive	81 (35.4)	19 (24.1)	193 (42.2)	1,585 (41.2)	**1,878 (40.7)**	**402 (34.8)**	<0.001
Negative	148 (64.6)	60 (75.9)	264 (57.8)	2,261 (58.8)	**2,733 (59.3)**	**752 (65.2)**	<0.001

**Abbreviations:** AI/AN = American Indian or Alaska Native; NH/OPI = Native Hawaiian or other Pacific Islander; PEP = postexposure prophylaxis.

* Mpox patients who reported symptom onset <14 days after their first JYNNEOS dose were presumed to have received PEP.

^†^ Mpox cases with missing or unknown hospitalization status were excluded from this analysis.

^§^ Pairwise chi-square testing was conducted between the included and excluded groups.

^¶^ Persons of Hispanic or Latino (Hispanic) origin might be of any race but are categorized as Hispanic; all racial groups are non-Hispanic.

Overall, 233 (5.0%) *Monkeypox* *virus* infections occurred in persons who received 1 JYNNEOS dose, 79 (1.7%) in those who received 2 doses, 457 (9.9%) in persons who received PEP,^¶^ and 3,845 (83.4%) in unvaccinated persons. A total of 250 (5.4%) mpox patients were hospitalized, including four (1.6%) who received 1 JYNNEOS dose, one (0.4%) who received 2 doses, 12 (4.8%) who received PEP, and 233 (93.2%) who were unvaccinated. Compared with unvaccinated mpox patients, the odds of hospitalization among persons with mpox who received 1 dose, 2 doses, and PEP were 0.27, 0.20, and 0.42, respectively (Table 2).

**TABLE 2:** Mpox hospitalizations, by JYNNEOS vaccination status (N = 4,611) — California, May 12, 2022–May 18, 2023

JYNNEOS vaccination status	Hospitalization status, no. (%)		Total, no.	OR (95% CI)
	Hospitalized n = 250	Not hospitalized n = 4,361		
Received 1 dose*	4 (1.7)	226 (98.3)	**230**	0.27 (0.08–0.65)
Received 2 doses^†^	1 (1.3)	78 (98.7)	**79**	0.20 (0.01–0.90)
Received PEP^§^	12 (2.6)	445 (97.4)	**457**	0.42 (0.22–0.72)
Unvaccinated^¶^	233 (6.1)	3,612 (93.9)	**3,845**	Ref

**Abbreviations:** OR = odds ratio; PEP = postexposure prophylaxis; Ref = referent group.

* Receipt of 1 JYNNEOS vaccine dose ≥14 days before their episode date (symptom onset date, specimen collection date, date of specimen receipt by the laboratory, or date laboratory report was received by the mpox surveillance registry, whichever was earlier), receipt of 2 doses with the second dose administered <14 days before the episode date, or receipt of 2 doses <24 days apart.

^†^ Receipt of 2 JYNNEOS vaccine doses ≥24 days apart with the second dose administered ≥14 days before the episode date.

^§^ Receipt of the first JYNNEOS vaccine dose after a known or suspected exposure and <14 days before the episode date.

^¶^ Receipt of no JYNNEOS vaccine doses.

Persons with HIV accounted for 1,878 (40.7%) of the 4,611 mpox patients included in the study and 140 (56.0%) of the 250 mpox hospitalizations (Table 3). Among hospitalized HIV-positive mpox patients, two of 81 (2.5%) had received 1 JYNNEOS dose, zero of 19 had received 2 doses, and seven of 193 (3.6%) had received PEP; 131 of 1,585 (8.3%) were unvaccinated. The odds of hospitalization among HIV-positive mpox patients who had received 1 dose of JYNNEOS or PEP were 0.28 and 0.42 times, respectively, the odds among unvaccinated HIV-positive mpox patients; no hospitalizations occurred among HIV-positive mpox patients who had received 2 JYNNEOS doses. Among HIV-negative mpox patients, the odds of hospitalization among 1-dose, 2-dose, and PEP recipients were 0.29, 0.36, and 0.41, respectively, times those among unvaccinated patients.

**TABLE 3:** Mpox hospitalizations, by vaccination status and HIV status* (N = 4,611) — California, May 12, 2022–May 18, 2023

HIV status/JYNNEOS vaccination status	Hospitalization status, no. (%)		Total, no.	OR (95% CI)
	Hospitalized n = 250	Not hospitalized n = 4,361		
**HIV positive (1,878)**				
1 dose^†^	2 (2.5)	79 (97.5)	**81**	0.28 (0.05–0.91)
2 doses^§^	0 (—)	19 (100.0)	**19**	0 (—)
PEP^¶^	7 (3.6)	186 (96.4)	**193**	0.42 (0.17–0.84)
Unvaccinated**	131 (8.3)	1,454 (91.7)	**1,585**	Ref
**HIV negative (2,733)**				
1 dose^†^	2 (1.4)	146 (98.6)	**148**	0.29 (0.05–0.93)
2 doses^§^	1 (1.7)	59 (98.3)	**60**	0.36 (0.02–1.65)
PEP^¶^	5 (1.9)	259 (98.1)	**264**	0.41 (0.14–0.91)
Unvaccinated**	102 (4.5)	2,159 (95.5)	**2,261**	Ref

**Abbreviations:** OR = odds ratio; PEP = post-exposure prophylaxis; Ref = referent group.

* HIV status was determined based on the presence or absence of a previous HIV case report submitted to the California Department of Public Health Office of AIDS.

^†^ Receipt of 1 JYNNEOS vaccine dose ≥14 days before episode date (symptom onset date, specimen collection date, date of specimen receipt by the laboratory, or date laboratory report was received by the mpox surveillance registry, whichever was earlier), receipt of 2 doses with the second dose administered <14 days before the episode date, or receipt of 2 doses <24 days apart.

^§^ Receipt of 2 JYNNEOS vaccine doses ≥24 days apart with the second dose administered ≥14 days before the episode date.

^¶^ Receipt of the first JYNNEOS vaccine dose after a known or suspected exposure and <14 days before the episode date.

** Receipt of no JYNNEOS vaccine doses.

## Discussion

During May 12, 2022–May 18, 2023, persons with mpox who had received 1 or 2 preexposure doses of JYNNEOS vaccine or had received JYNNEOS as PEP had lower odds of being hospitalized than did unvaccinated mpox patients. These findings suggest that receipt of both pre- and postexposure JYNNEOS vaccination reduces the odds of hospitalization among persons with mpox. In addition, among HIV-positive persons with mpox, who are at increased risk for severe mpox disease (*9*), those who were vaccinated had lower odds of hospitalization than did those who were unvaccinated. Zero hospitalizations were reported among persons infected with HIV who had received 2 doses of JYNNEOS vaccine. These findings underscore the benefit to persons with HIV infection of completing the 2-dose JYNNEOS vaccination series (*1*,*9*).

Approximately 300,000 doses of the JYNNEOS vaccine have been administered to California residents since May 26, 2022.** During this time, an estimated 64% of California’s at-risk population^††^ received 1 dose and 40% received 2 doses (*10*). Messaging to persons at higher risk for *Monkeypox* *virus* infection and persons with HIV infection should encourage completion of the 2-dose JYNNEOS vaccination series to limit virus transmission and mitigate disease severity.

Because JYNNEOS vaccine is not 100% effective, as more persons are vaccinated, the number of infections occurring in vaccinated persons will likely increase. It is important to prioritize further efforts to quantify the impact of JYNNEOS vaccination on disease severity (e.g., number of lesions, lesion spread, fever, and other prodromal signs and symptoms) to build upon current evidence. These efforts should be coupled with efforts to better understand the relationship between HIV and *Monkeypox* *virus*, including how viral suppression and CD4 counts might affect the immune response to mpox in response to vaccination; this could not be determined in the current study because of the limited number of infections that occurred among vaccinated persons with HIV infection.

### Limitations

The findings in this report are subject to at least six limitations. First, persons with mpox with symptom onset <14 days after receipt of the first JYNNEOS dose were presumed to have received PEP, but whether exposure preceded vaccination could not be confirmed. Second, differential misclassification of vaccination and hospitalization status, uncontrolled confounding, and selection bias might have affected this association. For example, these biases could manifest because the study did not determine whether persons in poorer health (and presumably higher a priori risk for hospitalization) were less likely to be vaccinated. If these persons were less likely to be vaccinated, the protective effect of vaccination might be overestimated, because it was not possible to control for baseline health status, especially among persons with HIV infection. Third, persons with diagnosed mpox, especially those who were vaccinated, might represent a population with better access to health care compared with persons with unreported mpox who were unvaccinated, leading to underestimation of the impact of JYNNEOS vaccination on prevention of mpox-associated hospitalization. Fourth, differential absence in hospitalization status with respect to vaccination status might have occurred, because the excluded population was less likely to report race and ethnicity, sexual orientation, or gender identity. If vaccinated persons were more likely to be misclassified as missing or not hospitalized, the protective effect would be overestimated. Fifth, because of the limited numbers of mpox cases and associated hospitalizations in persons who had received 2 JYNNEOS doses, the superiority of 2 doses versus 1 dose in preventing hospitalization among persons with mpox could not be demonstrated. Finally, associations by HIV status were limited by the low number of infections in vaccinated persons and resulted in wide CIs, perhaps because of the effectiveness of JYNNEOS at preventing *Monkeypox* *virus* infection.

### Implications for Public Health Practice

These findings provide evidence that JYNNEOS vaccination reduces the odds of hospitalization among persons with mpox. The protective effect of JYNNEOS was consistent among persons with mpox irrespective of HIV infection status. Although small case counts precluded determining whether 2 doses were superior to 1 dose in the total population, there were fewer hospitalizations among HIV-positive persons with mpox who had received 2 JYNNEOS doses compared with those who had received 1 dose, and in both groups, the odds of hospitalization were lower compared with those among unvaccinated persons. Persons at risk for mpox should receive the complete 2-dose JYNNEOS vaccine series to protect against infection and to reduce the odds of hospitalization if infection does occur.

## Footnotes

* Although a 28-day interval is recommended between the 2 JYNNEOS doses, a 4-day grace period allows for a minimum interval of 24 days between doses.

† The symptom onset date, specimen collection date, date of specimen receipt by the laboratory, or date of receipt of the laboratory report by the mpox surveillance registry, whichever occurred earlier.

§ 45 C.F.R. part 46.102(l)(2), 21 C.F.R. part 56; 42 U.S.C. Sect. 241(d); 5 U.S.C. Sect. 552a; 44 U.S.C. Sect. 3501 et seq.

¶ Median interval before episode date = 4 days; IQR = 1–7 days.

** Certain California residents received JYNNEOS vaccine before this date. May 26, 2022, was used for this analysis because this was the date that the California Department of Public Health had doses recorded among lots received by the Administration for Strategic Preparedness and Response for use as part of the state’s ongoing response to mpox.

†† Defined as the population recommended to receive the vaccine, which was estimated by CDC using 2021 data for men who have sex with men (MSM) with HIV preexposure prophylaxis indications and 2020 data for HIV prevalence among MSM from CDC AtlasPlus.
