# *Erratum:* Vol. 72, No. 36

**DOI:** 10.15585/mmwr.mm7310a4

**Published:** 2024-03-14

The report, “Reduced Odds of Mpox-Associated Hospitalization Among Persons Who Received JYNNEOS Vaccine — California, May 2022–May 2023” contained several errors.

On page 994, in Table 1, the total number of unvaccinated mpox cases should have read **n = 3,845.** Under the Gender identity characteristic, the p-value for the Female category should have read **0.122**, the p-value for the Genderqueer or non-binary category should have read **0.107**, the p-value for the Male category should have read **0.021**, the p-value for the Transgender female category should have read **0.058**, and the p-value for the Declined to answer category should have read **<0.001**. Under the Race and ethnicity characteristic, the p-value for the AI/AN category should have read **0.272**, the p-value for the Asian category should have read **0.644**, the p-value for the Black or African American category should have read **0.002**, the p-value for the NH/OPI category should have read **0.451**, and the p-value for the Multiple races category should have read **0.003**. Under the Sexual orientation characteristic, the p-value for the Declined to answer category should have read **0.009**, and the p-value for the Orientation not listed category should have read **0.057**.

